# Variations in Canine Behavioural Characteristics across Conventional Breed Clusters and Most Common Breed-Based Public Stereotypes

**DOI:** 10.3390/ani14182695

**Published:** 2024-09-17

**Authors:** Barbara Peťková, Lenka Skurková, Martin Florian, Monika Slivková, Zuzana Dudra Kasičová, Jana Kottferová

**Affiliations:** Workplace of Applied Ethology and Professional Ethics, Department of Public Veterinary Medicine and Animal Welfare, University of Veterinary Medicine and Pharmacy, Komenského 73, 04181 Košice, Slovakia

**Keywords:** dog, behaviour, dog personality, aggression, breed differences

## Abstract

**Simple Summary:**

The fascination with dog behaviour and public perceptions of breeds has been long-lasting. Media, literature, and individual experiences shape beliefs about breed behaviour. Most purebred dogs are now primarily kept as companions rather than for their original purposes. Through human selection, consistent behavioural traits have emerged in dog breeds. A questionnaire-based survey revealed that breeds perceived as aggressive are less aggressive than expected, while mixed-breeds tend to exhibit more aggression. The research proposes refraining from categorising specific breeds as dangerous and instead adopting a comprehensive approach. By focusing on education, training, selective breeding, and societal awareness, incidents involving dogs and humans can be minimised. Context and human influence play significant roles in dog aggression. Shifting away from breed stereotypes and utilising empirical evidence can enhance human-animal bonds and foster safer communities. Exploring genetic, environmental, and individual factors is essential for the implementation of effective dog training and management strategies.

**Abstract:**

Dog breeds are grouped based on scientific agreement, whether for traditional reasons or specific tasks during their domestication. Discrepancies may occur between public views of breed behaviour and actual evidence. This research aims to investigate differences in five behavioural traits (aggression towards people, aggression towards animals, fearfulness, responsiveness to training, and activity/excitability) by using the Dog Personality Questionnaire (DPQ) across six conventional groups/clusters of dog breeds (herding, hunting, guarding, companion dogs, potentially aggressive breeds, and mixed-breed dogs) and to assess hypotheses derived from common public presumptions. A cohort of 1309 dog owners sourced through diverse online platforms took part in the study. Contrary to stereotypes, the findings indicate that breeds labelled as “potentially aggressive” display lower levels of aggression compared to guarding breeds (χ^2^ (5) = 3.657, *p* = 0.041) and mixed-breeds (χ^2^ (5) = 3.870, *p* = 0.002). Additionally, mixed-breed dogs exhibited the highest levels of fearfulness among the six conventional clusters. In terms of aggression and gender, males demonstrated higher aggression levels towards both humans and animals compared to females (*p* = 0.001). These results challenge established assumptions and emphasise the necessity of evidence-based methodologies in the assessment of canine behaviour.

## 1. Introduction

The dog behaviour and public perception of individual breeds is one of the long-standing topics of interest not only to dog owners but also to the wider public. Along with the way dogs naturally fit into our homes and lives, ideas and presumptions about their personality and behaviour are emerging. The media, literature, and personal experience play a key role in this process, shaping our expectations and beliefs about how different breeds of dogs should behave [1,2,3]. But reality often reveals that our perceptions can be mispresented, and some ideas about our “furry companions” may be just myths [4].

In the modern sense, most of the purebred dogs serve as companions, not for their working ability or original breeding purpose [5,6,7]. During the domestication process, several reasons and motivations of humans have led to selective dog breeding, consequently resulting in distinct canine traits; some breeds excel at hunting, guarding, or herding, while others are ideal as pets and companions [8,9]. As a result of human (artificial) selection pressure, dog breeds exhibit diverse and consistent behavioural tendencies [8,10,11,12].

When discussing breed behavioural differences, there appears to be general agreement that dog breeds vary in behaviour or that purebred dogs have a specific behavioural adaptation [13]. In fact, *Canis lupus familiaris* is a subspecies that shows uncommon variation in morphological and behavioural phenotypes [8], and the substantial number of breeds makes studying breed-typical behaviour demanding [14]. 

To assess the suitability of dogs for breeding, working, and pet roles [15] or to determine the nature of behavioural traits and canine personalities across multiple breeds [7,16,17,18,19], questionnaires and psychometric approaches can be a viable alternative [20] to direct behavioural observations of large dog samples. The latter approach is time-consuming, logistically challenging, expensive, and complicated to evaluate [21]. Therefore, for estimating canine personality traits, in several studies [22,23,24,25] the focus turned to the surveys and to obtain the same type of data by the use of valid survey tools rating specific behavioural traits (e.g., aggressiveness, fear, etc.), usually completed by the owners, dog trainers, or veterinarians. To name a few, the Canine Behavioural Assessment and Research Questionnaire (C-BARQ(S)) [22,26], the Dog Personality Questionnaire (DPQ) [23], and the Monash Canine Personality Questionnaire (MCPQ(-R)) [24,25] are most commonly used, but other useful tools were utilised within the citizen science approach [27,28]. However, inconsistencies in the terminology used to describe the personality, temperament, and characteristic traits of dogs can lead to misunderstandings and challenges in understanding dog behaviour [29,30]. This ambiguity can defend communication and comparison of research findings, potentially impacting the generalisability and applicability of studies on dog behaviour. Therefore, establishing clear and standardised definitions for these terms is crucial for enhancing our comprehension of dog behaviour and advancing research in this area [30].

The differences in behavioural tendencies or personality traits among various dog breeds or groups/clusters of dog breeds formed on conventions or genetic relatedness were described in a few studies [12,14,17,31,32,33,34,35,36,37]. The personality research focusing on the behavioural profile or some trait(s) of specific breeds (such as German Shepherds, Labrador retrievers, Golden retrievers, Hungarian Vizsla, Czechoslovakian wolfdogs, or others) is covered only by a limited number of studies [38,39,40,41,42,43]. Studies looking into breed variations in canine behaviour validated the presence of distinctions both among and within breeds [8,44,45,46]. Therefore, caution is advised in the interpretation of personality traits across dog breeds, as highlighted by Mirkó et al. [41] and Wells [47], due to the potential impact of external factors on their genetic foundations and resultant individual differences. Actually, variability within a breed can be even greater than among breeds [3,37,48].

Moreover, investigations and studies grounded on data provided by canine owners frequently uncover that specific breeds are inaccurately associated with certain traits, while other traits are underestimated—especially in relation to aggression [17,49,50]. Clarke et al. [50] conducted research that validated the theory of *acculturation* or *contact hypothesis*, determining that the stereotyping and generalisations of breeds in dogs mirror the phenomenon of racism in humans—the extent and nature of interaction with dog breeds significantly shape the predisposition towards making unfair judgements about them. Dog breed stereotypes are pervasive [51], despite the scientific evidence for greater variations within a breed than among breeds [3,8,43]. In addition, results suggest that people’s perceptions of dog breeds can be influenced not only by verbal and visual representations [52] but also by veterinary education and experience shape beliefs about dog breeds—ratings for feelings of “warmth” and “trust” towards specific dog breeds were lower in veterinary academic respondents compared to the public and undergraduates [51].

The disparity between public perception and the actual behaviour of dogs can result in numerous significant implications. Not only does idealising certain breeds result in unrealistic expectations and miscommunication with dogs [53], but also negative prejudices and stereotypes about dog breeds lead to discrimination or issues with legislation [54,55]. Certain dog breeds have a historical association with dog fighting or guarding, which contributes to their negative perception among the public, e.g., the enduring negative perception of Pitbull terriers due to their background in dog fighting [56]. Similarly, in the study of Clarke et al. [57] focused on the anticipated level of aggressiveness in various dog breeds, respondents over five times more likely indicated Staffordshire Bull terriers as dangerous by image alone.

Moreover, some dog breeds are labelled as “dangerous” or “potentially dangerous,” leading to specific legal repercussions. Dog breed-specific legislation (BSL) has been established in different countries, such as the UK and the USA, to address issues concerning dog aggression and bites. Breed-specific legislation (BSL) pertains to laws that regulate or prohibit the ownership of certain breeds considered to present a heightened risk of aggression or harm. However, research suggests varying opinions regarding its efficacy. Studies have shown that while some people believe certain breeds are predisposed to aggression, there is a lack of consistency in findings regarding breed-specific risk [43,58]. Furthermore, investigations into the impact of BSL on reducing dog bite injuries have revealed limited effectiveness, with enforcement measures like muzzles and leash laws also showing minimal impact [59]. An in-depth analysis of selected European countries where regulations oversee the breeding standards of certain breeds can be found in the Supplementary Material (Table S1).

Therefore, the aim of our study was to find out whether the common stereotyped statements about the characteristics of dogs (owner perceived) coincide with the individual statements of the sample of Czech and Slovak respondents. We addressed our aim by objective to determine variations in aggression (and exploring potential risk factor–sex), fearfulness, and trainability among different breed group clusters and to compare the results with five common stereotypes with little, inconsistent, or even no scientific support, thus either confirming or refuting these prevailing beliefs; (1) potentially dangerous dog breeds”, or “dangerous dog breeds” are more aggressive than the other breeds, both towards people and (2) animals, (3) spayed female dogs are more aggressive than intact ones regardless of the breed (higher likelihood of human-directed aggression and reactive behaviour, e.g., in [60,61,62,63], (4) mix-breeds are more fearful than other dog breeds’ categories (an increased risk of noise phobia, dog-directed and human-directed fear, or sensitivity to touch have been reported in mix-breeds e.g., in the studies of Blackwell et al. [64], Schneider et al. [65] and Temesi and (5) guarding breeds are most responsive to training e.g., [66,67].

## 2. Materials and Methods

### 2.1. Ethical Statement

Data were collected from Slovak and Czech dog owners via an online questionnaire. Participants voluntarily and anonymously completed the questionnaires, ensuring that their privacy rights were not compromised (no personally identifiable information was collected). The introductory letter of the questionnaires contained information regarding informed consent for the data to be used for scientific purposes.

Ethical review and approval were deemed unnecessary for the animal study as it involved the use of an online questionnaire to gather data on dog demographics and owner-reported dog personality. In accordance with the prevailing legislation in Slovakia, the collection of non-invasive observational data on dog demographics and behaviour does not fall under the category of animal experimentation. Therefore, it can be conducted without seeking specific authorisation from the Ethical Committee of the University of Veterinary Medicine and Pharmacy in Kosice.

### 2.2. Study Design and Data Collection

Researchers collected data through an online survey, where dog owners assessed their pets’ behavioural characteristics. Participants from Slovakia and Czechia responded to five demographic items and seventy-five statements about their dogs’ reactions in everyday situations as part of the Dog Personality Questionnaire (DPQ) [23]. Besides the statements about canine behaviour in general, included in the DPQ, the following demographic data about the dogs were collected: age (in months), sex, breed, status intact/neutered, pedigree (Y/N), housing conditions (dog kept only indoors, indoors/outdoors, only outdoors).

The survey was available for the owner/breeders online for 10 months and promoted through dog-related social media (e.g., Facebook) groups, forums, and breeders. At the beginning of the survey, respondents were informed about the study and granted informed consent for the scientific use of the data. To maintain statistical independence, they evaluated only one dog, preferably a well-known one, if they had multiple pets.

### 2.3. Dog Personality Questionnaire (DPQ)

For the objectives of this study, the Dog Personality Questionnaire (DPQ), a reliable and valuable tool, was utilised to acquire a standardised dataset [23]. The Dog Personality Questionnaire (DPQ) demonstrated satisfactory levels of inter-rater reliability and test-retest reliability and showed a significant correlation with a behavioural test battery conducted by kennel personnel. The DPQ is available in two formats: a 45-item short form and a 75-item long form. The DPQ assesses dogs across five personality factors, which consist of several facets, which serve as more specific subcategories reflecting different aspects of each personality trait: Factor 1, fearfulness (fear of people, non-social fear, fear of dogs, fear of handling); Factor 2, aggression towards people (general aggression, situational aggression); Factor 3, activity/excitability (excitability, playfulness, active engagement, companionability); Factor 4, responsiveness to training (trainability, controllability); and Factor 5, aggression towards animals (aggression towards dogs, prey drive, dominance over other dogs). The study opted for the longer form to maximise data collection, especially for additional research purposes. The questionnaire details of the items used are listed in Supplementary Material in Table S2. The scores for each factor, as assessed in the questionnaire, were analysed in accordance with established guidelines and a scoring key, detailed in Supplementary Material in Table S3.

### 2.4. Clustering the Dog Breeds

Breeds of dogs were categorised into six groups: guarding breeds, herding breeds, companion breeds, hound breeds, mix-breeds, and potentially aggressive breeds. Clustering the dog breeds into groups was based on similarities among breeds regarding their original function, the perception of various breeds in the public, and partly on the status of modern breeds of dogs (Table 1). The description of breed-group typical behavioural profiles (outlined in Supplementary Material Table S4) significantly influences our understanding of different dog breeds and shapes our expectations regarding their behaviour. Detailed descriptive data about the categories of the dog breed clusters included in the study with the list of breeds is available in the Supplementary Material (Table S5).

### 2.5. Statistical Analyses

Participants in the study evaluated the dogs’ behaviour using 75 statements, rating their agreement on a 7-point scale from 1 to 7 for each statement. A score of 1 denoted “strongly disagree,” while 7 signified “strongly agree,” with 4 representing a neutral stance of “neither agree nor disagree.” Higher scores indicate a stronger expression of the corresponding trait. While most items were directly summed into factors, a few were reverse-coded before factor creation, as per the scoring instructions. 

Scores for each factor (fearfulness: fear of people, non-social fear, fear of dogs, fear of handling; aggression towards people: general aggression, situational aggression; activity/excitability: excitability, playfulness, active engagement, companionability; responsiveness to training: trainability, controllability; and aggression towards animals) were analysed in accordance as per published guidelines/scoring key (please refer to Table S3). 

Test selection depends on data normality; therefore, non-parametric statistical techniques have been chosen for examining differences in dog behaviour: Independent-samples Mann–Whitney U test and Independent-samples Kruskal–Wallis H test. 

To examine variations in aggression, fearfulness, and trainability between different breed group clusters and explore risk factors for species-specific breed aggression (sex differences in aggression towards people and animals), Kruskal–Wallis H-test was performed with the assumption of homogenous variance between groups, and Mann–Whitney U test was performed to determine significant sex differences in aggression. 

Pairwise comparisons were done afterwards to find out which groups of dog breeds differ in terms of aggression towards animals and people, fearfulness, and trainability. 

Kruskall–Wallis ANOVA test for independent samples was performed to test the impact of housing conditions (outdoor, indoor, and outdoor/indoor) in relation to the evaluated behavioural traits—aggressiveness towards animals and aggressiveness towards people, fearfulness, and trainability. 

For the statistical analyses, the SPSS statistical program (version 21.0) was used.

## 3. Results

### 3.1. Demography

In total, the questionnaire data were recorded from 1309 owners with various breeds, including mix-breeds. In terms of the breed group, the dogs were assigned to six groups: companion breeds (N = 347), hound breeds (N = 151), herding breeds (N = 185), guarding breeds (N = 215), mix breeds (N = 168), and potentially aggressive breeds (N = 243). Out of them, 537 (41%) were males and 772 (59%) were females. The mean age (±S.D.) of the dogs in the sample was 56.76 ± 38.96 months. Additionally, 1093 (83.5%) dogs out of 1309 were intact, and 216 (16.5%) were neutered (20.6% of females and 10.8% of males); 474 dogs out of 1309 (36.2%) spent most of their time in indoor housing conditions, 454 dogs (34.7%) were mostly kept in outdoor conditions, and 381 dogs (29.1%) were mostly kept both indoor and outdoor conditions. Finally, 824 dogs (62.9%) were with pedigree, and 485 dogs (37.10%) were without pedigree. Basic demographic data about the study subjects are presented in Table 2.

### 3.2. Variations in Aggression, Fearfulness, and Trainability between Different Breed Group Clusters

Besides the variations in some of the behavioural traits among six breed groups, we evaluated several hypotheses summarising the most common prejudices in the public about behavioural traits (aggressiveness, fearfulness, or trainability) of different categories of dog breeds and the perception of these categories in modern society. The hypothesis reflecting the concept of “potentially dangerous dog breeds” was also included. Descriptive statistical data are presented in Table 3.

#### 3.2.1. Differences among Breed Categories in Aggressiveness towards People and Animals

**Figure 1:**
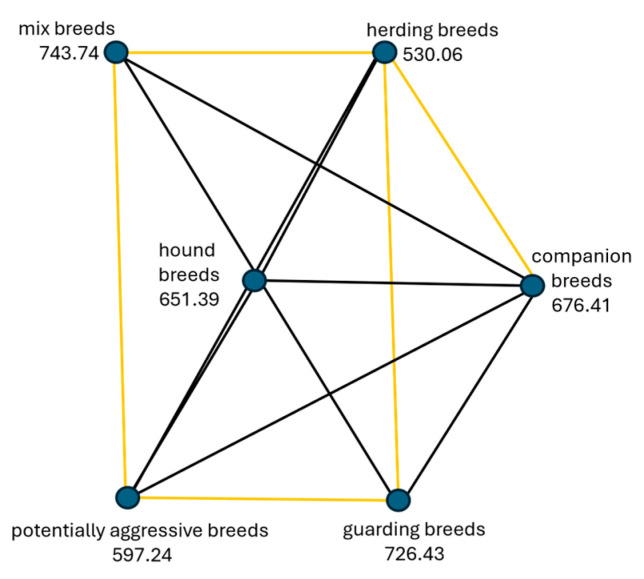
Pairwise comparison for various categories of dog breeds of aggression towards people. Regarding the potentially aggressive dogs, statistically significant results (yellow lines) showed they are less aggressive towards people than the category of guarding (*p* = 0.004) or mix-breeds (*p* = 0.002). No statistically significant differences between categories of dog breeds are represented by black lines.

The highest levels of aggressiveness towards people were reported by the owners in the category of mix-breeds, followed by guarding breeds, companion breeds, hound breeds, and potentially aggressive breeds. The category of herding dog breeds was the least aggressive towards people (Figure 1). The first tested hypothesis (H1a) “Aggression towards people is the same across categories of dog breeds” was rejected because the Sig. value (*p*-value) of 0.001 is less than the *p*-value of 0.05 selected for the test as the level of significance.

Post-hoc test results show significant differences between pairs of independent variables (categories of dog breeds). Significant differences were between herding dog breeds (as least aggressive) and companion dog breeds χ^2^(5) = 4.261, *p* = 0.001, guarding dog breeds χ^2^(5) = 5.190, *p* = 0.001, and the category of mix-breeds of dogs χ^2^(5) = 5.315, *p* = 0.001. Also, statistically significant differences were between the category of potentially aggressive dogs and more aggressive categories, such as guarding dog breeds χ^2^(5) = 3.657, *p* = 0.004, and mix-breeds of dogs χ^2^(5) = 3.870, *p* = 0.002. Detailed post-hoc analysis is available in the pairwise comparison table in Supplementary Material (Table S7). Statistically significant differences between categories of dog breeds are represented by yellow lines (Figure 1).

The analysis conducted aimed to investigate the relationship between housing conditions and levels of aggressiveness towards people. The results indicated that there were no statistically significant differences in aggressiveness based on the different housing conditions assessed (χ^2^(2) = 4.9026, *p* = 0.0862).

**Figure 2:**
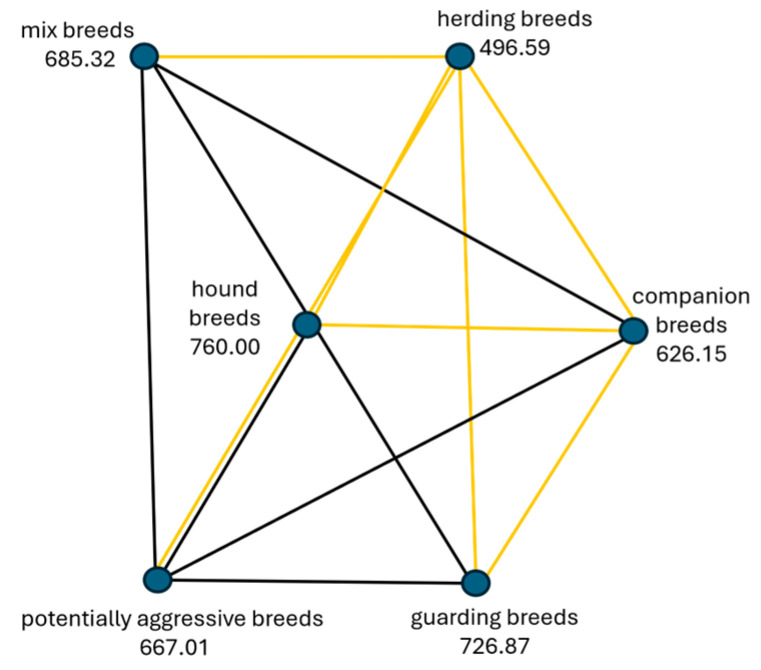
Pairwise comparison for various categories of dog breeds of aggression towards animals. Statistically significant differences between categories of dog breeds are represented by yellow lines, where the potentially aggressive dog breeds were more aggressive towards the animals than herding dogs (*p* = 0.001). No statistically significant differences between categories of dog breeds are represented by black lines.

The second hypothesis (H1b), “Aggression towards animals is the same across categories of dog breeds”, was rejected. The Sig. value (*p*-value) of 0.001 is less than the *p*-value of 0.05 selected for the test as the level of significance. The highest levels of aggressiveness towards animals were reported by the owners in the category of hound breeds, followed by guarding breeds, mix-breeds, potentially aggressive breeds, and companion breeds. Less aggressive towards animals was the category of herding dog breeds (Figure 2).

**Figure 3:**
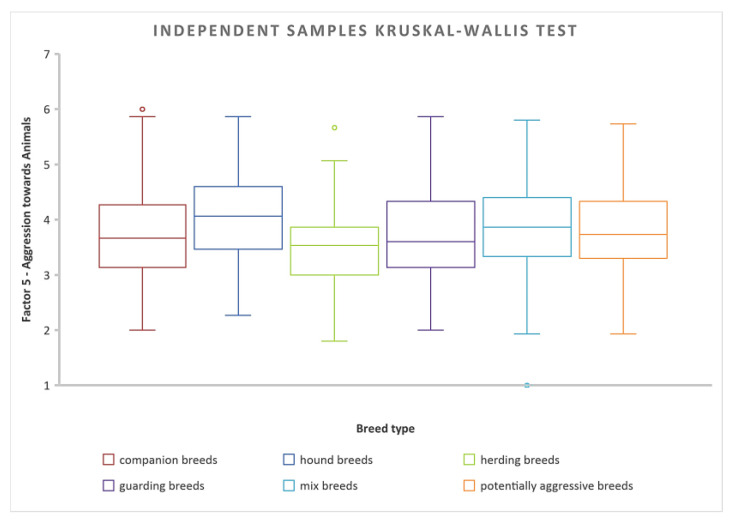
Statistical analysis showed that there was a statistically significant difference in aggression towards animals score between the potentially aggressive breeds and herding breeds, χ^2^(5) = 4.622, *p* = 0.001.

Post-hoc test results show significant differences between pairs of independent variables (categories of dog breeds). Significant differences were between herding dog breeds (as least aggressive) and every other category: companion dog breeds χ^2^(5) = 3.766, *p* = 0.002, potentially aggressive dog breeds χ^2^(5) = 4.622, *p* = 0.001, mix-breeds χ^2^(5) = 4.686, *p* = 0.001, guarding dog breeds χ^2^(5) = 6.077, *p* = 0.001, and category of hound breeds χ^2^(5) = 6.356, *p* = 0.001. Also statistically significant were differences between the categories of companion breeds and guarding breeds χ^2^(5) = 3.071, *p* = 0.032, and hound breeds χ^2^(5) = 3.633, *p* = 0.004. Detailed post-hoc analysis is available in the pairwise comparison table in Supplementary Material (Table S8). Independent-samples Kruskal–Wallis H test was applied to determine differences in aggression towards animals within different breed categories (Figure 3).

Additionally, the results indicated that there were no statistically significant variations in aggressive behaviour towards animals when comparing different housing conditions (χ^2^(2) = 0.6206180, *p* = 0.7332). 

#### 3.2.2. Differences among Breed Categories in Fearfulness

**Figure 4:**
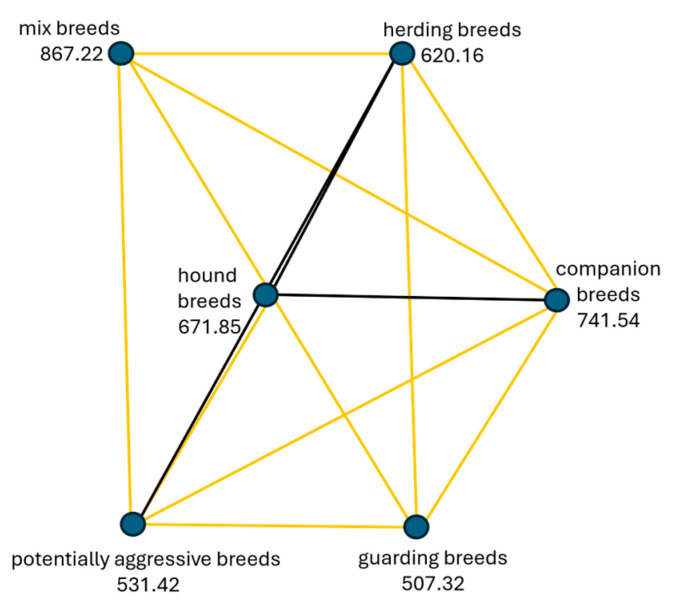
Pair-wise comparison of *fearfulness* in different breed categories. Statistically significant results (yellow lines) showed that mix breeds are more fearful than potentially aggressive breeds (*p* = 0.001), hound breeds (*p* = 0.001), companion breeds (*p* = 0.006), guarding breeds (*p* = 0.001), and herding breeds (*p* = 0.001). No statistically significant differences between categories of dog breeds are represented by black lines.

The hypothesis (H2) “Fearfulness is the same across categories of dog breeds” was rejected because the Sig. value (*p*-value) of 0.001 is less than the *p*-value of 0.05 that we were using for our test. The post-hoc test results (detailed post-hoc analysis is available in the pairwise comparison table in Supplementary Material; Table S9) reveal a significant difference between pairs of independent variables. Specifically, this indicates that there exists a notable variation in fearfulness across various categories of dog breeds. In summary, the highest levels of fearfulness were reported by the owners in the category of mix-breeds, followed by companion dogs, hound dogs, herding dogs, and potentially aggressive dogs. The least fearful was the category of guarding dog breeds (Figure 4).

**Figure 5:**
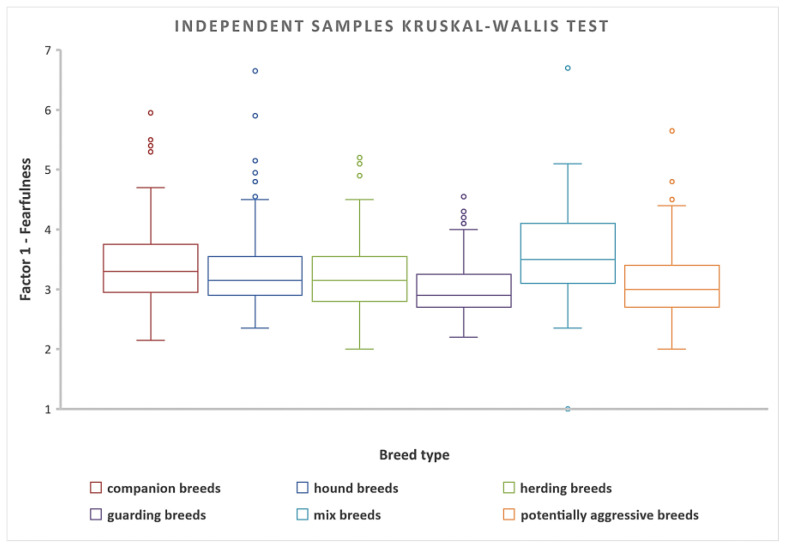
Independent-Samples Kruskal–Wallis Test for fearfulness in various categories of dog breeds.

The analysis revealed no statistically significant differences in levels of fearfulness associated with the different housing conditions (χ^2^(2) = 2.499, p = 0.2867). When examining mix-breeds of dogs (which tend to be the most fearful), the statistical analysis revealed notable differences in fearful reactions compared to other breed types. Mix-breeds were more fearful than potentially aggressive breeds χ^2^(5) = 8.857, *p* = 0.001, hound breeds χ^2^(5) = 4.611, *p* = 0.001, companion breeds χ^2^(5) = 3.539, *p* = 0.006, guarding breeds χ^2^(5) = 9.250, *p* = 0.001, and herding breeds χ^2^(5) = 6.135, *p* = 0.001. Independent-samples Kruskal–Wallis H test was applied to determine differences in fearfulness within different breed categories (Figure 5).

#### 3.2.3. Differences among Breed Categories in Responsiveness to Training

**Figure 6:**
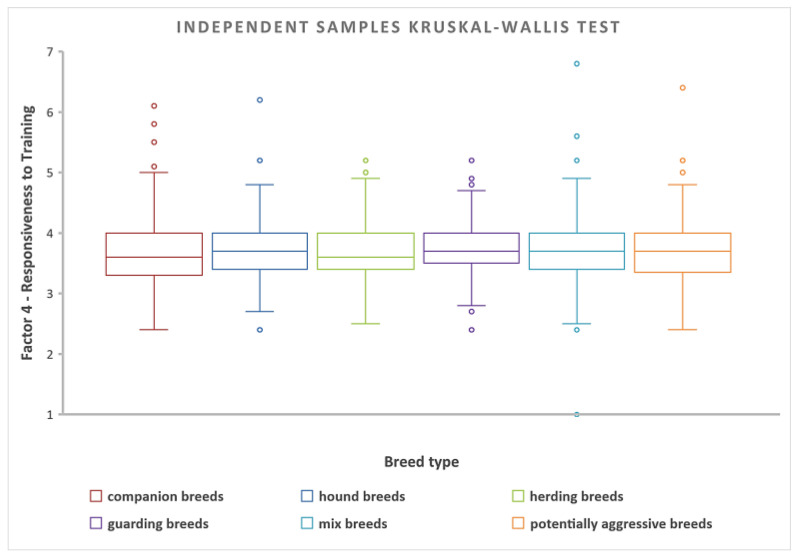
Independent-Samples Kruskal–Wallis Test for *responsiveness to training* in various categories of dog breeds.

The hypothesis (H3), “Trainability/Responsiveness to training varies across categories of dog breeds”, was rejected. No statistically significant differences in responsiveness to training were found based on dogs’ breed χ^2^(5) = 7.144, *p* = 0.210 (Figure 6). Multiple comparisons were not performed because the overall test does not show significant differences across samples (*p* = 0.210, non-significant). Moreover, the analysis showed that there were no statistically significant differences in trainability related to the various housing conditions (χ^2^(2) = 0.9818, *p* = 0.6121).

### 3.3. Sex Differences in Dogs in Aggressiveness towards People and Animals

**Figure 7:**
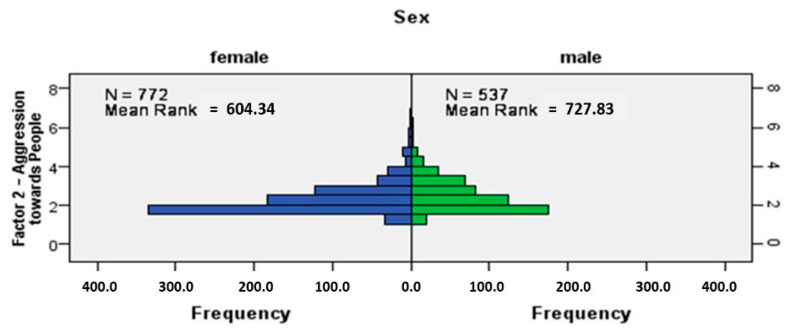
Independent-samples Mann–Whitney U test (*p*-value 0.05, significance level): Comparison of the aggressiveness towards people in dog males and females–frequency histogram quantifying the frequency of observations from one group ranking higher than those from another (comparison of distributions).

The hypotheses (H4a and H4b) “Aggression towards people is the same across categories of sex” and “Aggression towards animals is the same across categories of sex” were rejected because the Sig. value (*p*-value) of 0.001 was less than the *p*-value of 0.05 selected for the test as the level of significance. Independent-samples Mann–Whitney U test was applied and showed there is a significant difference between male and female dogs in aggression towards people, U = 168,171.000, z = 5.825, *p* = 0.001. Males were significantly more aggressive towards people (mean rank 727.83) in comparison to female dogs (mean rank 604.34) (Figure 7). 

**Figure 8:**
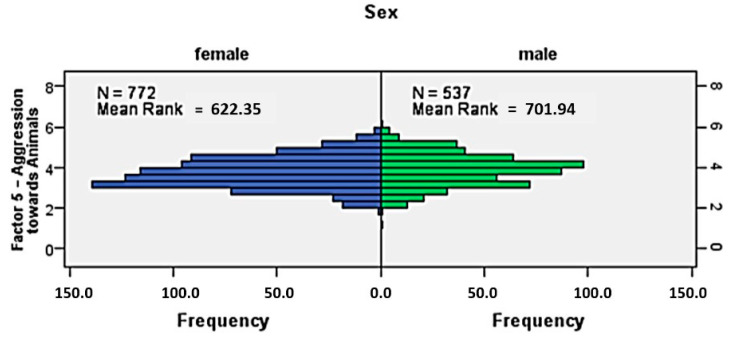
Independent-samples Mann–Whitney U test (*p*-value 0.05, significance level): Comparison of the aggressiveness towards animals in dog males and females–frequency histogram quantifying the frequency of observations from one group ranking higher than those from another (comparison of distributions).

Moreover, male dogs were also significantly more aggressive (mean rank 701.94) towards animals in comparison to female dogs (mean rank 622.35) U = 182 075.000, z = 3.748, *p* = 0.001 (Figure 8).

Independent-samples Mann–Whitney U test was applied also to test potential distinctions in aggression between spayed and not spayed female dogs. When testing the hypotheses (H5a), “Aggression of females towards people is the same across categories of neutered/intact status”, and (H5b), “Aggression of females towards animals is the same across categories of neutered/intact status”, no statistically significant differences in aggression towards people U = 48,784.500, z = 0.020, *p* = 0.984 nor in aggression towards animals were found, U = 46,737.500, z = 0.797, *p* = 0.425.

### 3.4. Stereotypes Associated with Breed-Specific Behaviour

Comparing the results based on the DPQ in variations in aggression, fearfulness, and trainability with the most common stereotypes associated with canine breed-specific behaviour, we found out that only the stereotype related to higher fearfulness of mixed-breeds corresponds to the findings presented in the section above. Beliefs (or stereotypes) about “dangerous dog breeds” perceived by the public as more aggressive than the other breeds, or more aggressive females than males (or neutered than intact ones), or guarding breeds as the most responsive to training are not supported by the results of our DPQ survey.

## 4. Discussion

### 4.1. Variations in Aggression, Fearfulness, and Trainability between Different Breed Group Clusters

Differences in aggression, fearfulness, and trainability between different breed groups point to diverse behavioural profiles within the canine population. Our study based on the owner-reported canine behaviour reveals significant differences, with some breed groups showing higher levels of aggression towards humans or animals, while others showed lower timidity or greater trainability. Understanding these differences is critical not only in dog training but also in developing dog ownership laws. It is critical that these go beyond common-breed stereotypes.

#### 4.1.1. Variations in Aggressiveness Levels towards People and Animals across Different Breed Categories

Our study revealed notable differences in the frequency of aggression reported by owners towards both humans and animals across various dog categories, challenging the initial hypothesis that proposed a consistent level of aggression among different dog breeds. Mixed-breeds and guarding breeds showed the highest rates of aggression towards humans while herding breeds showed the lowest rates of aggression. These results align with prior studies suggesting that genetic predispositions and historical functions of breeds play a significant role in shaping their aggressive tendencies [31,32]. Due to their innate protective instincts, guarding breeds, traditionally bred to protect property and livestock, exhibit more aggressive behaviour. The perpetuation of this innate trait through generations of selective breeding has led to escalated aggression levels [55,68,69]. 

Research conducted by Casey et al. [70] proposes that aggression frequently emerges as a learned response to circumstances rather than being a universal trait among individuals. Advancing age in dogs may constitute a risk factor, as evidenced by its association with an elevated likelihood of aggression towards unfamiliar individuals in both indoor and outdoor settings. Their study also identified hound dogs as posing a potential aggression risk towards household members. It is essential to acknowledge that the variables measured only accounted for a minimal proportion of the variability (<10%) observed between aggressive and non-aggressive animals, suggesting that factors unique to individual dog experiences have a more substantial impact on the development of aggression. These findings imply that while principal characteristics of dogs and their owners may influence aggression at a population level, it is inappropriate to extrapolate assumptions regarding aggression risk in individual animals based solely on breed characteristics.

Conversely, our results present a challenge to prevailing stereotypes that categorise specific dog breeds as inherently predisposed to aggression. The research indicated that breeds frequently subjected to societal stigma and negative media representation as “potentially aggressive” did not demonstrate the highest levels of aggression towards humans or other animals. This disparity indicates that societal perceptions do not consistently correspond with objective evidence. The stigmatisation of breeds as more dangerous may arise from isolated occurrences or historical prejudices rather than a holistic comprehension of the breed’s typical behaviours [55,71].

#### 4.1.2. Variations in Fearfulness across Different Breed Categories

The analysis of fear responses across different dog breed categories revealed remarkable disparities, notably with mix-breeds displaying the highest levels of fearfulness. The heightened fearfulness observed in mix-breeds could potentially be attributed to their diverse genetic backgrounds and potentially more complex early life encounters [72]. Dogs in shelters or those lacking a clear historical background are more predisposed to encounter adverse and stressful circumstances, resulting in escalated fear responses and subsequent behavioural challenges [73,74].

Guarding breeds demonstrated the lowest level of fearfulness, in accordance with their traditional duties that require confident and stable temperaments. Svartberg [32] categorised these breeds based on variations in behaviour, specifically emphasising low fearfulness, to guarantee their effectiveness in fulfilling their tasks. This intentional selection for specific behavioural traits highlights the significant influence of genetic factors on the development of fear-related behaviours.

Companion breeds, conversely, displayed moderate levels of fearfulness, which could potentially stem from their selective breeding for characteristics that are favourable for cohabitation with humans, where an excessive degree of fear would not be advantageous. Individuals often choose companion breeds due to their friendly nature and calm dispositions, which make them ideal for domestic life and close human interaction [75]. Regardless, there is variability observed within this group, emphasising the notable influence of individual encounters and training in shaping responses to fear [76].

The observation that mix-breed dogs exhibited a notably higher level of fear compared to various other breed categories, including potentially aggressive breeds, hound breeds, companion breeds, guarding breeds, and herding breeds, implies that fear-related behaviours are greatly influenced by early life encounters and socialisation. This discovery is in accordance with existing literature that highlights how fearfulness in canines frequently stems from insufficient socialisation and exposure to diverse settings during critical developmental stages [76,77,78]. Therefore, the management of fearfulness in dogs requires a comprehensive approach that prioritises positive early interactions, proper socialisation, and ongoing behavioural guidance.

#### 4.1.3. Variations in Responsiveness to Training across Different Breed Categories

The findings deviated from our initial expectations, as there was a lack of significant differences in the responsiveness to training among various categories of dog breeds. The result indicates that breeds might share the ability for training equally, a concept that challenges previous assumptions. The implementation of positive reinforcement training techniques, which have proven to be effective despite the genetic predispositions of various breeds, could be responsible for the lack of substantial variability observed [79,80]. This presents a challenge to the stereotype that specific breeds, such as those bred for guarding (or working) purposes, inherently possess higher trainability due to their historical functions.

The data implies that the differences among individuals within breeds often exceed the breed averages in terms of trainability, which is in line with the findings of earlier studies [23,50,81,82,83]. The impact of individual characteristics and training methods employed appears to have a more considerable influence on trainability, despite the potential impact of breed-specific traits on certain behavioural aspects. Utilising positive reinforcement strategies, which concentrate on reinforcing desirable behaviours, can effectively improve trainability across various breeds, underscoring the significance of employing ethical and scientifically validated training approaches [84]. 

Our findings highlight the importance of considering the individual dog’s disposition, educational background, and the trainer’s approach when evaluating trainability [31,85]. This perspective suggests that relying on assumptions regarding breeds for training suitability may be misguided and counterproductive. When considering the results of studies, the robustness of the human-canine connection and the consistency in training methodologies, as opposed to solely the dog’s breed, have a substantial effect on training efficacy [86,87]. This highlights the importance of positive interactions and training routines in improving dogs’ responsiveness to training.

### 4.2. Sex Variations in Dogs in Aggressiveness towards People and Animals

Sex differences in aggression were significant, with male dogs exhibiting higher levels of aggression towards both individuals and animals in comparison to female dogs. This observation aligns with existing literature on sexual dimorphism in canine behaviour, attributing such variances to hormonal influences and sex-specific socialisation patterns. The impact of testosterone on aggressive behaviours is well documented, potentially explaining the increased aggression in male dogs [88]. Correspondingly, the study by Goodloe and Borchelt [89] reported similar patterns. Moreover, an investigation by Takeuchi et al. [90] illustrated that male dogs were predisposed to facing issues linked to aggression targeted at owners but not towards strangers. Pérez-Guisado and Muñoz-Serrano [91] further identified males as exhibiting elevated levels of dominance aggression in comparison to females, although their study did not explore other forms of aggression. 

To summarise, our investigation and previous studies imply that the difference between male and female dogs in owner-directed aggression is more obvious and easily identifiable than differences in other forms of aggression. Furthermore, societal and owner expectations frequently influence distinct handling and training strategies for male and female dogs, potentially affecting these behavioural distinctions [82,92].

Within certain contexts, individuals could choose to neuter or spay to address dog aggression; nevertheless, the studies have generated conflicting outcomes, e.g., [93,94,95]. Research has demonstrated a lack of significant variation in levels of aggression [49,96], whereas other studies have suggested an increase in aggression among neutered or spayed dogs [97,98]. Pérez-Guisado and Muñoz-Serrano [91] clarified the connection between the process and the display of dominance aggression in male (decreased) and female (increased) dogs. Significantly, it revealed a lack of substantial variance in aggression levels between spayed and intact female dogs. 

Conversely, a recent investigation by Kolkmeyer et al. [99] analysed the behavioural connections between neutering and breed, particularly emphasising huskies and bulldogs. The results indicated that castrated males from both breeds displayed higher levels of aggression towards humans compared to their intact counterparts, showing distinct variations in aggression towards other dogs and stress-related behaviours depending on neuter status and breed.

These results present a challenge to established beliefs regarding the correlation between spaying and increased aggression in female dogs, indicating that the role of reproductive status in shaping aggressive tendencies may be less significant than previously proposed [53]. This aligns with studies indicating that alterations in behaviour following spaying are predominantly influenced by individual disposition and external circumstances rather than solely hormonal variations [100].

### 4.3. Stereotypes Linked to Behaviour Specific to Certain Breeds

Our study highlights the importance of utilising empirical data to challenge societal perceptions and stereotypes related to different dog breeds. This awareness possesses the potential to enhance the efficacy of managing, educating, and decision-making processes regarding dog ownership and legislation specific to breeds [50,53,55,72].

The significant differences in aggression, fearfulness, and trainability across various breed categories highlight the complex interaction among genetics, environment, and personal interactions in influencing the behaviour of dogs. Addressing behavioural issues in dogs requires a comprehensive approach that considers genetic predispositions, individual differences, and environmental factors rather than relying on simplistic and potentially misleading breed stereotypes. Understanding that mix-breed dogs could demonstrate increased fearfulness because of their miscellaneous backgrounds and shelter experiences can support the creation of specific interventions to meet their individual needs [73].

Our findings suggest that it would be beneficial for public education campaigns and policies to highlight the importance of proper socialisation, responsible dog ownership, and training methods rather than focusing on restrictions based on specific breeds. Misconceptions surrounding behaviours specific to breeds possess the potential to induce unjustified concerns and discriminatory actions, which may not effectively address the underlying issues related to aggression and fearfulness in dogs [12,55,83]. Furthermore, there is a growing demand for further exploration of alternative strategies, such as public education concerning animal behaviour and more strict regulations on leashes, to enhance public safety without needing to implement restrictions that target specific breeds [101].

The ethical and legal implications of breed-specific legislation (BSL) have been studied, with suggestions that addressing breeding practices and ownership regulations may be more effective in promoting responsible dog ownership and reducing dog-related incidents [43,58,102]. 

Genetic, hormonal, and environmental factors influence the intricate nature of aggressive behaviour in dogs. It’s important to focus on each dog’s unique characteristics and specific context rather than relying solely on breed stereotypes or assumptions related to gender and reproductive health. In conclusion, this nuanced comprehension of aggression has the potential to contribute to the development of more efficient approaches for the management and reduction of aggressive or fearful tendencies in dogs, thus improving the safety and well-being of both dogs and humans alike.

### 4.4. Limitations of the Study

However, we must admit that our study has some limitations, highly relevant for cautious interpretations of our results. First, the behavioural data were obtained using the self-reporting questionnaire (DPQ), which is a method often used in behavioural studies both in humans and animals. The disadvantage of this method of obtaining data includes the possibility of providing invalid answers and not answering truthfully, especially on questions that respondents perceive as “sensitive” questions—they have tendencies to respond in a more acceptable or expected way (so-called social desirability bias). Another issue of self-reporting studies is a response bias, or the individual’s inclination to respond in a certain way regardless of the topic or question, which affects the reliability and validity of studies based on the use of questionnaires. Some other issues include the questionable clarity of the items to the respondents or lack of flexibility [103].

The second limitation we would like to address is that our study evaluated only the factor of breed type (plus variable “sex”). We did not collect nor statistically analyse the additional data about the history of dogs, socialisation or experiences, which would be highly relevant, especially regarding canine aggression or fearfulness. 

Development and variations in those traits are multifactorial and may be related not only to breed type but could be also a function of current or past living conditions, age, or some other factors. Those factors (increasing or decreasing the level of fear or aggression) can be defined as risks [104]. The category of companion-related risk (the biological characteristics of participants) includes breed (evaluated in our study), size, age, gender, neutering, and health status. The category of socialisation-related risk (the social (in)experience of the participants) involves the lack of early socialisation and issues around hierarchy, interpersonal relationships in the group, or inappropriate social experiences. Lastly, the situational risk is the context or circumstances of the situation in which the aggressive attacks occur (predatory, territorial, fear-based). Finally, it is important to remember the possible interaction between those three categories of risk factors, e.g., the risk of socialisation might depend on the biological features of the companions [105]. According to Miklósi [104], all three types of risks can and should be identified for both humans and animals, although there is a bias in the literature emphasising the dog’s side of companion-related risks. 

The benefits of outdoor housing, especially in encouraging increased activity levels, should be considered alongside the unique traits of individual dogs and the overall quality of the outdoor environment. Dogs do not react the same way to these settings; instead, their ability to adapt to different housing conditions is greatly affected by factors such as their previous experiences and natural temperament. Research indicates that the aspect of “outdoor living” may be a contributing factor to the increased occurrence of behavioural issues in dogs, as well as a higher prevalence of aggression [41,106,107] among those who spend more time outdoors.

Potential explanations for this phenomenon may include reduced interaction with humans compared to dogs that live indoors, insufficient mental or physical stimulation leading to frustration or anxiety, and inadequate training. Additionally, territorial aggression appears to be more frequently observed in these dogs.

Nevertheless, research conducted in Czechia and Slovakia [108,109] has not identified significant disparities in aggression levels between dogs that are housed outdoors and those that are kept indoors, nor between rural and urban settings. It can be speculated that “outdoor living,” along with factors such as urbanisation and the rural-urban living comparison, is not an isolated determining factor; rather, it is likely a multifaceted issue. The aggression levels of dogs living outdoors may be influenced by the quality of the human-dog relationship, the degree of human proximity to the animal, the owner’s awareness regarding training and the dog’s need for mental and physical engagement, the daily routines of both the dog and the owner, and other related factors.

Focusing on the aim of our study to challenge the prevalent stereotypes, we argue that a complex perspective and multifactorial approach to this topic are crucial.

## 5. Conclusions

The utilisation of questionnaire-based data presents a significant opportunity for further analysis of behavioural traits across different dog breeds. Our survey supports previous findings, revealing that breeds often classified as potentially aggressive or dangerous exhibit less aggression in many aspects compared to other breeds. Notably, mixed-breed dogs display the highest levels of aggressive behaviour across various categories.

Addressing the frequently debated issue of identifying specific breeds as dangerous, we argue that such categorisation is unjustified. Instead, a comprehensive approach that includes public education, training, selective breeding for appropriate temperaments based on future individual use, and promoting social tolerance toward dangerous animals aims not to isolate them from society but to enhance their social interactions and skills. This multifaceted strategy contributes to reducing incidents of attacks and sudden accidents involving both humans and dogs. However, mismanaged conflicts can still result in serious harm, especially when dealing with large, unbalanced dogs. Aggression in dogs is context-dependent and often subject to human influence. This study provides valuable insights into canine behaviour by dispelling myths and offering evidence-based information. Through these findings, we can promote responsible dog ownership, eliminate breed-specific regulations with legislation focused more on overall responsible dog ownership regardless of breed, and encourage safer interactions between dogs and humans.

In conclusion, our research emphasises the need to move beyond breed-based stereotypes. By focusing on empirical data and evidence-based practices, we can improve canine welfare and strengthen the human-animal bond, creating safer and more harmonious communities. To achieve more effective and humane dog training and management strategies, continued exploration of the complex interactions between genetic, environmental, and individual factors shaping dog behaviour remains essential.

## Figures and Tables

**Table 1:** Conventional categorisation of dog breeds into six categories with respect to their original function, use, and status in modern society (a detailed list of breeds included in the study is available in Supplementary Material, Table S5).

Breed Category Title	Description and Historical Function	Number of Breeds	Number of Individuals	%	Characteristic Behavioural Traits	Examples of Breeds Represented in the Research
Companion breeds	Companionship	42	347	27	High sociability (friendly), loyal	Cavalier King Charles Spaniel, Maltese, Poodle, Yorkshire terrier
Mix-breeds	Non-specified breed, unknown origin, mixture of two or more breeds	-	168	13	Diverse traits, higher prevalence of fearfulness, aggressiveness, etc.	Mixture of various breeds, often living feral and reproducing freely, or living in shelters
Hound (hunting) breeds	Hunting of prey	29	151	12	Excellent scent, alert, active	Basset hound, Beagle, Dachshund, Weimaraner
Guarding breeds	Bred for work (police dogs, etc.)	7	215	16	High trainability, alert, intelligent, protective	Belgian shepherd dog, German shepherd dog, Hovawart
Herding breeds	Driving livestock, used for control of other animals	18	185	14	Trainability, intelligence, herding ability, protective	Australian Shepherd, Bernese Mountain dog, Border collie, Shetland sheepdog, Slovakian Cuvac
Potentially aggressive breeds *	Category designed according to the legislation	19	243	19	Higher aggressiveness, resistance, boldness *	American Staffordshire terrier, Bullmastiff, Doberman, English Bull terrier, Rottweiler
**Summary**		**115**	**1309**	**100**		

* With respect to the breed, dogs considered to be potentially dangerous included those who fulfilled the majority or all of the following characteristics: (1) powerful character and big bravery; (2) strong musculature, athletic physique, and resistance; (3) standard weight >20 kg; (4) large and robust head with deep mouth. Some of these criteria are not supported by scientific data, but they are based on many legislations of the European Union and include the breeds most frequently referred to as dangerous by the lay public in our country (a list of the dog breeds included in the category of potentially aggressive breeds for the purpose of our study is available in the Supplementary Material Table S6).

**Table 2:** Basic demographic data about the subjects of the study (dogs included in the survey).

Basic Characteristics of the Study Subjects					
Sex	n	%	Neutered Status	n	%
Male	537	41.00	Yes	216	16.50
Female	772	59.00	No	1093	83.50
**Housing** **conditions**	**n**	**%**	**Pedigree status**	**n**	**%**
Only indoors	474	36.20	Yes	824	62.90
Only outdoors	454	34.70	No	485	37.10
Indoors and outdoors	381	29.10	**Total number of dogs**	1309	100

**Table 3:** Descriptive statistical data for all the categories of dog breeds included in the study (total numbers, mean values, and standard deviation) for behavioural categories according to the DPQ.

Category of Dog Breeds		Fearfulness	Aggression Towards People	Responsiveness to Training	Aggression Towards Animals
Companion breeds (N = 347)	Mean	3.3885	2.3802	3.6746	3.7291
	SD	0.6179	0.9008	0.5561	0.7545
Hound breeds (N = 151)	Mean	3.2967	2.2987	3.7046	3.9815
	SD	0.6478	0.8136	0.5252	0.7358
Herding breeds (N = 185)	Mean	3.1846	2.0373	3.6724	3.4771
	SD	0.5378	0.5730	0.4425	0.6674
Guarding breeds (N = 215)	Mean	3.0337	2.4033	3.7642	3.9451
	SD	0.4525	0.7972	0.4683	0.7462
Mix-breeds (N = 168)	Mean	3.6024	2.5232	3.6970	3.8341
	SD	0.6865	0.9571	0.6073	0.7952
Potentially aggressive breeds (N = 243)	Mean	3.0593	2.1877	3.6815	3.8156
	SD	0.5145	0.7713	0.5126	0.7431

SD = standard deviation, N = total number of dogs.

## Data Availability

The original contributions presented in the study are included in the article and supplementary materials. The raw data supporting the conclusions of this article will be made available by the authors on request.

## Supplementary Materials

The following supporting information is available online at https://www.mdpi.com/article/10.3390/ani14182695/s1, Table S1: Regulations governing breeding conditions for selected breeds in chosen European countries; Table S2: Dog Personality Questionnaire (Jones, 2008); Table S3: Scoring key for Dog Personality Questionnaire; Table S4: Description of breed-group typical behavioural profiles; Table S5: List of breeds included in each of the categories of dog breeds; Table S6: List of breeds included in the group, potentially aggressive breeds; Table S7: Pairwise comparison table for the H1a (aggression towards people); Table S8: Pairwise comparison table for the H1b (aggression towards animals); Table S9: Pairwise comparison table for the H2 (fearfulness).
