# Supplementary material for: Variations in Canine Behavioural Characteristics across Conventional Breed Clusters and Most Common Breed-Based Public Stereotypes
Source: Animals (Basel). 2024 Sep 17;14(18):2695. doi: 10.3390/ani14182695 (PMC11429495; doi:10.3390/ani14182695)
Supplement: Supplementary file 1 [file animals-14-02695-s001.zip › Table S3 Scoring key for Dog Personality Questionnaire (DPQ).pdf]

Table S3: Scoring key for Dog Personality Questionnaire (DPQ)

Factor<br>Facet	Item number on long form
**Factor 1 – Fearfulness**	
Facet 1 – Fear of People	**R1, 12,** 30, **47,** 54
Facet 2 – Nonsocial Fear	**6, R19,** 24, **R38,** R58
Facet 3 – Fear of Dogs	R9, **21, 36,** 66, **70**
Facet 4 – Fear of Handling	**28,** 32,42, **61, 74**
**Factor 2 – Aggression towards People**	
Facet 1 – General Aggression	**13,** 23, **R33, 68,** 73
Facet 2 – Situational Aggression	2, 17, **43, 51, 62**
**Factor 3 – Activity/Excitability**	
Facet 1 – Excitability	**27, 53,** 55, **R69**, 72
Facet 2 – Playfulness	R3, **R16, 31,** 46, **59**
Facet 3 – Active Engagement	**R10**, 14, **25, 40,** 48
Facet 4 – Companionability	7, **35, R44, 63,** 67
**Factor 4 – Responsiveness to Training**	
Facet 1 – Trainability	37, R45, **R50, R64, 71**
Facet 2 – Controllability	R4, **11, R18,** R29, **56**
**Factor 5 – Aggression towards Animals**	
Facet 1 – Aggression towards Dogs	**5,** 8, **R34,** 57, **R60**
Facet 2 – Prey Drive	**15,** 22, 26, **39, 65**
Facet 3 – Dominance over Other Dogs	**20,** 41, **R49,** 52, **75**

Explanatory note: An R in front a item indicates that the item is reverse coded. Bolded item numbers indicate items that also appear on the short form of the DPQ, though numbered differently.
