# Supplementary material for: Variations in Canine Behavioural Characteristics across Conventional Breed Clusters and Most Common Breed-Based Public Stereotypes
Source: Animals (Basel). 2024 Sep 17;14(18):2695. doi: 10.3390/ani14182695 (PMC11429495; doi:10.3390/ani14182695)
Supplement: Supplementary file 1 [file animals-14-02695-s001.zip › Table S4 Description of breed-group typical behavioural profiles.pdf]

Table S4: Description of breed-group typical behavioural profiles according to their public perception with respect to their original function/use and status in the modern society.

Dog breed groups	Typical behavioural breed profile
**Companion breeds**	A companion dog is primarily a pet, serving the essential role of providing companionship to humans. These dogs are also known as emotional support animals, aiding people with mental health disabilities in managing their symptoms. While most dogs can fulfil this role, including working dog breeds like retrievers, toy dog breeds are often kept purely for the pleasure of their company rather than as working animals. Smaller breeds are typically chosen for their convenience as pets. In modern Western cultures, dogs are considered family members or valued companions, and their original working roles have largely diminished. Unlike in the past, where behavioural traits were crucial, today's breed standards prioritize other factors.
**Mix breeds**	In the case of mixed-breed dogs, discussing common typical characteristics becomes challenging. Unlike purebred breeds that adhere to specific standards, mixed breeds exhibit diverse behavioural traits.
**Hound breeds**	Hound dogs have a rich history as skilled hunters, and their abilities vary based on whether they primarily rely on scent or sight. Hound dogs come in various shapes and sizes and making generalizations about hounds is challenging due to the diverse range of breeds within the group but they all share a passion for the hunt. Whether they follow scents or rely on their keen eyesight, these dignified and trustworthy companions thrive on exercise and purpose.
**Guarding breeds**	Guardian dogs possess remarkable protective instincts, and their loyalty and fearlessness make them excellent companions for those seeking security. Proper training and socialization are crucial to ensure they channel their strength and instincts appropriately. These fiercely loyal breeds form strong bonds and are dedicated protectors. Fearlessly confronting potential threats and intruders, they are often muscular and robust. Their physical strength allows them to effectively deter or subdue intruders. With an innate sense of vigilance, guardian dogs remain alert and attentive, constantly monitoring their surroundings. Instinctively, they protect their territory, family members, and property, skilfully differentiating between normal visitors and potential dangers.
**Herding breeds**	Herding breeds were specifically developed to assist humans in herding livestock. These breeds required agility, stamina, and innate herding instincts. Additionally, their coats were designed to be functional, enabling them to adapt to various climates and terrains. These dogs work closely with human shepherds and exhibit natural intelligence and responsiveness, making them highly trainable. They share an instinctual ability to control the movement of other animals, making them excellent companions for herding tasks.
**Potentially aggressive breeds**	In terms of breed characteristics, dogs considered potentially dangerous exhibit several common traits. These include a powerful character and great bravery. They also appear to be bred for fighting or to have the characteristics of types bred for that purpose. Additionally, they have well-developed musculature, athletic bodies, and resilience. Typically weighing more than 20 kilograms, these dogs often have large, robust heads with deep mouths. It's important to note that while some of these criteria lack scientific backing, they are frequently referenced in various European Union legislations as indicators of potentially dangerous breeds according to public perception.
