# Supplementary material for: Variations in Canine Behavioural Characteristics across Conventional Breed Clusters and Most Common Breed-Based Public Stereotypes
Source: Animals (Basel). 2024 Sep 17;14(18):2695. doi: 10.3390/ani14182695 (PMC11429495; doi:10.3390/ani14182695)
Supplement: Supplementary file 1 [file animals-14-02695-s001.zip › Table S5 List of the breeds included into each of the categories of dog breeds (FCI breed nomenclature was used; n=1.309).pdf]

Table S5: List of the breeds included into each of the categories of dog breeds (FCI nomenclature is provided in the table; n=1.309).

**Categorisation of dog breeds**: breed and total number of dogs included in the study
**Mix-breed dogs (n=168):** crossbreeds (168)
**Guarding breeds (n=215):** Belgian Shepherd Dog (27), Berger de Beauce (6), German Shepherd Dog (144), Giant Schnauzer (3), Hovawart (29), Dutch Shepherd Dog (5), Russian Black Terrier (1)
**Hunting breeds (n=151):** Hungarian Short-haired Pointer (Viszla) (3), English Setter (1), Gordon Setter (4), Bedlington Terrier (1), Border Terrier (2), Scottish Terrier (1), Continental Toy Spaniel (4), Welsh Terrier (6), German Wire-haired Pointing Dog (2), Weimaraner (27), Kleiner Münsterländer (1), German Hunting Terrier (1), German Short-haired Pointing Dog (5), Irish Red Setter (3), Flat Coated Retriever (6), English Springer Spaniel (3), Welsh Springer Spaniel (1), Dachshund (32), Beagle (17), Basset Hound (7), Hanoverian Scenthound (1), Bavarian Mountain Scenthound (3), Hungarian Wire-haired Pointer (5), Slovakian Hound (2), Bohemian Wire-haired Pointing Griffon (1), Nova Scotia Duck Tolling Retriever (2), Nederlandse Kooikerhondje (2), Wirehaired Slovakian Pointer (1)
**Herding breeds (n=185):** Old English Sheepdog (5), Bernese Mountain Dog (13), Appenzell Cattle Dog (2), Entlebuch Cattle Dog (3), Great Swiss Mountain Dog (6), Shetland Sheepdog (11), Pyrenean Mountain Dog (1), Slovakian Chuvach (8), Collie Rough (3), Polish Lowland Sheepdog (1), Bearded Collie (2), Lapponian Herder (1), Australian Cattle Dog (1), Collie Smooth (3), Border Collie (81), Czechoslovakian Wolfdog (17), Australian Shepherd (13), White Swiss Shepherd Dog (14)
**Companion breeds (347):** White Swiss Shepherd Dog (15), Fox Terrier (smooth) (1), Welsh Corgi (2), Newfoundland (4), Maltese (18), West Higland White Terrier (9), Yorkshire Terrier (28), German Spitz (8), French Bulldog (3), Golden Retriever (23), Labrador Retriever (56), Cavalier King Charles Spaniel (16), Boston Terrier (2), Bulldog (2), Dalmatian (6), Irish Wolfhound (1), Whippet (1), American Cocker Spaniel (14), Fox Terrier (wire) (10), Poodle (22), Schnauzer (6), Miniature Schnauzer (23), Miniature Pinscher (5), Borzoi-Russian Hunting Sighthound (3), Bolognese (2), Italian Greyhound (1), Pekingese (1), Shih Tzu (3), Samoyed (3), Bichon Frise (5), Chihuahua (14), Lhasa Apso (3), Afgan Hound (2), Pug (2), Shiba (4), Norfolk Terrier (1), Cotton de Tulear (1), Chinese Crested Dog (3), Azawakh (1), Peruvian Hairless Dog (1), Parson Russell Terrier (3), Jack Russell Terrier (19)

Potentially aggressive breeds (243): English Bull Terrier (23), Staffordshire Bull Terrier (27), Dogue de Bordeaux (6), Dobermann (18), Rhodesian Ridgeback (17), Rottweiler (16), Bullmastiff (5), Fila Brasileiro (10), Perro de Presa Mallorquin (2), Akita Inu (15), Tosa Inu (6), American Staffordshire Terrier (30), Dogo Argentino (17), Caucasian Shepherd Dog (3), Central Asian Shepherd Dog (5), Cane Corso (17), Dogo Canario (3), Pitbull Terrier* (22), American Bulldog* (1)

** Pitbull terrier and American bulldog are breeds non-recognized by FCI.*
