# Supplementary material for: Variations in Canine Behavioural Characteristics across Conventional Breed Clusters and Most Common Breed-Based Public Stereotypes
Source: Animals (Basel). 2024 Sep 17;14(18):2695. doi: 10.3390/ani14182695 (PMC11429495; doi:10.3390/ani14182695)
Supplement: Supplementary file 1 [file animals-14-02695-s001.zip › Table S6 List of the breeds included into the category „potentially aggressive breeds “.pdf]

Table S6: List of the breeds included into the category „*potentially aggressive breeds* “(n=243).

	FCI number*	FCI breed nomenclature*	Total number
**Potentially aggressive breeds**	**11**	English Bull Terrier	23
	**76**	Staffordshire Bull Terrier	27
	**116**	Dogue de Bordeaux	6
	**143**	Dobermann	18
	**146**	Rhodesian ridgeback	17
	**147**	Rottweiler	16
	**157**	Bullmastiff	5
	**225**	Fila Brasileiro	10
	**249**	Perro de Presa Mallorquin	2
	**255**	Akita	15
	**260**	Tosa	6
	**286**	American Staffordshire Terrier	30
	**292**	Dogo Argentino	17
	**328**	Caucasian Shepherd Dog	3
	**335**	Central Asian Shepherd Dog	5
	**343**	Cane Corso	17
	**346**	Dogo Canario	3
	**non-recognized by FCI**	Pitbull Terrier	22
	**non-recognized by FCI**	American Bulldog	1
		**Total**	**243**

*Explanatory note: The FCI classification (number and nomenclature) is provided in the table solely for the purpose of better orientation.
