# Supplementary material for: Variations in Canine Behavioural Characteristics across Conventional Breed Clusters and Most Common Breed-Based Public Stereotypes
Source: Animals (Basel). 2024 Sep 17;14(18):2695. doi: 10.3390/ani14182695 (PMC11429495; doi:10.3390/ani14182695)
Supplement: Supplementary file 1 [file animals-14-02695-s001.zip › Table S7 Pairwise comparison table for the H1a (aggression towards people).pdf]

Table S7: Pairwise comparison table for the H1 (aggression towards people).

Sample1-Sample2	Test Statistic	Std. Error	Std. Test Statistic	Sig.	Adj. Sig.
**Herding breeds-potentially aggressive breeds**	-67.181	36.812	-1.825	.068	1.000
**Herding breeds-hound breeds**	121.325	41.376	2.932	.003	.050
**Herding breeds-companion breeds**	146.344	34.345	4.261	.000	.000
**Herding breeds-guarding breeds**	-196.366	37.834	-5.190	.000	.000
**Herding breeds-mix breeds**	-213.682	40.207	-5.315	.000	.000
**Potentially aggressive breeds-hound breeds**	54.145	39.094	1.385	.166	1.000
**Potentially aggressive breeds-companion breeds**	79.164	31.558	2.508	.012	.182
**Potentially aggressive breeds-guarding breeds**	129.185	35.324	3.657	.000	.004
**Potentially aggressive breeds-mix breeds**	146.501	37.855	3.870	.000	.002
**Hound breeds-companion breeds**	25.019	36.781	.680	.496	1.000
**Hound breeds-guarding breeds**	-75.040	40.058	-1.873	.061	.915
**Hound breeds-mix breeds**	-92.357	42.307	-2.183	.029	.436
**Companion breeds-guarding breeds**	-50.022	32.745	-1.528	.127	1.000
**Companion breeds-mix breeds**	-67.338	35.460	-1.899	.058	.864
**Guarding breeds-mix breeds**	-17.316	38.849	-.0446	.656	1.000

Each row tests the null hypothesis that the Sample 1 and Sample 2 distributions are the same. Asymptotic significances (2-sided tests) are displayed. The significance level is .05.

*Explanatory note: If the p-value in the row with adjusted significance is less or equal to the p value selected for the test as a level of significance (p = 0.05) means significant difference between the categories of dog breeds.
