# Supplementary material for: Variations in Canine Behavioural Characteristics across Conventional Breed Clusters and Most Common Breed-Based Public Stereotypes
Source: Animals (Basel). 2024 Sep 17;14(18):2695. doi: 10.3390/ani14182695 (PMC11429495; doi:10.3390/ani14182695)
Supplement: Supplementary file 1 [file animals-14-02695-s001.zip › Table S8 Pairwise comparison table for the H1b (aggression towards animals).pdf]

Table S8: Pairwise comparison table for the H1b (aggression towards animals).

Sample1-Sample2	Test Statistic	Std. Error	Std. Test Statistic	Sig.	Adj. Sig.
**Herding breeds-companion breeds**	129.558	34.400	3.766	.000	.002
**Herding breeds- potentially aggressive breeds**	-170.419	36.871	-4.622	.000	.000
**Herding breeds-mix breeds**	-188.732	40.272	-4.686	.000	.000
**Herding breeds-guarding breeds**	-230.278	37.895	-6.077	.000	.000
**Herding breeds-hound breeds**	263.407	41.443	6.356	.000	.000
**Companion breeds- potentially aggressive breeds**	-40.461	31.609	-1.293	.196	1.000
**Companion breeds-mix breeds**	-59.174	35.517	-1.666	.096	1.000
**Companion breeds-guarding breeds**	-100.720	32.798	-3.071	.002	.032
**Companion breeds-hound breeds**	-133.850	36.840	-3633	.000	.004
**Potentially aggressive breeds-mix breeds**	18.313	37.916	.483	.629	1.000
**Potentially aggressive breeds-guarding breeds**	59.859	35.381	1.692	.091	1.000
**Potentially aggressive breeds-hound breeds**	92.988	39.157	2.375	.018	.263
**Mix breeds-guarding breeds**	41.546	38.912	1.068	.286	1.000
**Mix breeds-hound breeds**	74.675	42.375	1.762	.078	1.000
**Guarding breeds-hound breeds**	33.129	40.123	.826	.409	1.000

Each row tests the null hypothesis that the Sample 1 and Sample 2 distributions are the same. Asymptotic significances (2-sided tests) are displayed. The significance level is .05.

Explanatory note: If the p-value in the row with adjusted significance (Adj. Sig. = significance with Bonferroni correction to reduce the risk of errors (or false-positives)) is less or equal to the p value selected for the test as a level of significance (p = 0.05) means significant difference between the categories of dog breeds.
