# Supplementary material for: Variations in Canine Behavioural Characteristics across Conventional Breed Clusters and Most Common Breed-Based Public Stereotypes
Source: Animals (Basel). 2024 Sep 17;14(18):2695. doi: 10.3390/ani14182695 (PMC11429495; doi:10.3390/ani14182695)
Supplement: Supplementary file 1 [file animals-14-02695-s001.zip › Table S9 Pairwise comparison table for the H2 (fearfulness).pdf]

Table S9: Pairwise comparison table for the H2 (fearfulness).

Sample1-Sample2	Test Statistic	Std. Error	Std. Test Statistic	Sig.	Adj. Sig.
**Guarding breeds-Potentially aggressive breeds**	-24.095	35.378	-.681	.049	1.000
**Guarding breeds-Herding breeds**	112.844	37.892	2.978	.003	.044
**Guarding breeds-Hound breeds**	164.530	40.120	4.101	.000	.001
**Guarding breeds-Companion breeds**	234.217	32.795	7.142	.000	.000
**Guarding breeds-Mix breeds**	-359.902	38.909	-9.250	.000	.000
**Potentially aggressive breeds-Herding breeds**	88.749	36.869	2.407	.016	.241
**Potentially aggressive breeds-Hound breeds**	140.435	39.154	3.587	.000	.005
**Potentially aggressive breeds-Companion breeds**	210.122	31.607	6.648	.000	.000
**Potentially aggressive breeds -Mix breeds**	335.808	37.913	8.857	.000	.000
**Herding breeds-Hound breeds**	51.686	41.440	1.247	.212	1.000
**Herding breeds-Companion breeds**	121.373	34.398	3.529	.000	.006
**Herding breeds-Mix breeds**	-247.058	40.269	-6.135	.000	.000
**Hound breeds-Companion breeds**	69.686	36.837	1.892	.059	.878
**Hound breeds-Mix breeds**	-195.372	42.372	-4.611	.000	.000
**Companion breeds-Mix breeds**	-125.686	35.515	-3.539	.000	.006

Each row tests the null hypothesis that the Sample 1 and Sample 2 distributions are the same. Asymptotic significances (2-sided tests) are displayed. The significance level is .05.

*Explanatory note: If the p-value in the row with adjusted significance is less or equal to the p value selected for the test as a level of significance (p = 0.05) means significant difference between the categories of dog breeds.
