# Supplementary material for: Variations in Canine Behavioural Characteristics across Conventional Breed Clusters and Most Common Breed-Based Public Stereotypes
Source: Animals (Basel). 2024 Sep 17;14(18):2695. doi: 10.3390/ani14182695 (PMC11429495; doi:10.3390/ani14182695)
Supplement: Supplementary file 1 [file animals-14-02695-s001.zip › Table S1 Regulations governing breeding conditions for selected breeds in chosen European countries.pdf]

Table S1: Regulations governing breeding conditions for selected breeds in chosen European countries.

	Dog breed	European country [status]
**Potentially aggressive breeds**	English Bull terrier	Austria[1](Vienna, Lower Austria, Vorarlberg), Cyprus[2], Denmark[1], Germany[2], Ireland[1], Iceland[2], Norway[1], Spain[1], Switzerland[1](varies by canton), Ukraine[1]
	Staffordshire Bull terrier	Austria[1](Vienna, Lower Austria, Vorarlberg), Denmark[1], France[1], Germany[2], Iceland[2], Ireland[1], Lithuania[1], Portugal[1], Romania[1], Spain[1], Switzerland[2], Ukraine[1]
	Dogue de Bordeaux	Austria[1](Vorarlberg), Denmark[1], Germany[1] (Baden-Württemberg, Bavaria, Brandenburg, Hamburg), Switzerland[1](varies by canton), Ukraine[1] (before November 10, 2021)
	Dobermann	Germany[1](Brandenburg), Ireland[1], Romania[1], Spain[1](Valencia), Switzerland[1](varies by canton), Ukraine[1]
	Rhodesian Ridgeback	Austria[1](Vorarlberg), Ireland[1], Ukraine[1]
	Rottweiler	Austria[1](Vienna, Lower Austria), France[1], Germany[1](Bavaria, Brandenburg, Hamburg, Hesse), Ireland[1], Poland[2], Portugal[1], Romania[1], Spain[1], Switzerland[1](varies by canton), Ukraine[1]
	Bullmastiff	Austria[1](Vienna, Vorarlberg), Denmark[1], Germany[1] (Baden-Württemberg, Bavaria, Brandenburg, Hamburg, Hesse), Ireland[1], Romania[1], Spain[1](Valencia), Switzerland[1](varies by canton), Ukraine[1]
	Fila Brasileiro	Austria[1](Vienna, Vorarlberg), Cyprus[2], Denmark[2], Germany[1] (Baden-Württemberg, Bavaria, Brandenburg, Hamburg), Iceland[2], Lithuania[1], Norway[2], Portugal[1], Romania[1], Spain[1], Switzerland[1](varies by canton), United Kingdom[2]
	Perro de Presa Mallorquin	Germany[1](Bavaria, Brandenburg), Poland[2]
	Akita Inu	Ireland[1], Spain[1], Ukraine[1]
	Tosa Inu	Austria[1](Vienna, Lower Austria, Vorarlberg), Cyprus[2], Denmark[2], France[1], Germany[2], Iceland[2], Ireland[1], Norway[2], Poland[2], Portugal[1], Romania[1], Spain[1], Switzerland[1](varies by canton), Ukraine[1], United Kingdom[2]
	American Staffordshire terrier	Austria[1](Vienna, Lower Austria, Vorarlberg), Cyprus[2], Denmark[2], France[1], Iceland[2], Norway[2], Poland[2], Portugal[1], Romania[1], Spain[1], Switzerland[1](varies by canton), Ukraine[1], United Kingdom[2]
	Dogo Argentino	Austria[1](Vienna, Lower Austria, Vorarlberg), Cyprus[2], Denmark[2], Germany[2], Iceland[2], Lithuania[1], Norway[2], Poland[2], Portugal[1], Romania[1], Spain[1], Switzerland[1](varies by canton), Ukraine[1], United Kingdom[2]
	Caucasian Shepherd Dog	Denmark[2], Germany[1] (Hamburg, Hesse), Lithuania[1], Poland[2], Romania[1], Ukraine[1]
	Central Asian Shepherd Dog	Denmark[2], Ukraine[1]
	Cane Corso	Denmark[1], Germany[1] (Bavaria, Brandenburg), Romania[1], Switzerland[1](varies by canton), Ukraine[1]
	Dogo Canario	Denmark[1], Germany[1] (Bavaria, Brandenburg), Poland[2], Romania[1], Switzerland[1](varies by canton), Ukraine[1]
	Pitbull Terrier	Austria[1](Vienna), Cyprus[2], Denmark[2], France[1], Germany[2], Iceland[2], Ireland[1], Lithuania[2], Norway[2], Poland[2], Portugal[1], Romania[2], Spain[1], Switzerland[1](varies by canton), United Kingdom[2], Ukraine[1]
	American Bulldog	Denmark[2], Germany[1] (Bavaria, Hesse), Lithuania[1], Poland[2], Ukraine[1], United Kingdom[2]

*Explanatory note: [1] (*restricted)* means that there are specific rules or regulations governing the ownership, breeding, or import of that particular breed within that country, these restrictions can vary widely depending on specific country, [2] (*banned*) means that the breed is prohibited or not allowed within a specific country, region, or locality, bans are typically put in place due to concerns about public safety, aggressive behaviour, or incidents involving specific breeds.

Sources:

https://www.petraveller.com.au/blog/tag/banned-breeds

Austria: https://www.oesterreich.gv.at/themen/reisen_und_freizeit/haustiere/1/2.html

Cyprus: https://www.moa.gov.cy/moa/vs/vs.nsf/vs07_en/F7E307422C0B1B99C22575350040DAE2?OpenDocument

Denmark: https://en.foedevarestyrelsen.dk/animals/animal-welfare/danish-legislation-on-dogs

France: https://www.petraveller.com.au/blog/banned-dog-breeds-in-france

Germany:https://www.zoll.de/EN/Private-individuals/Travel/Entering-Germany/Restrictions/Dangerous-dogs/provisions_imposed_by_individual_federal_states.html?nn=201960&faqCalledDoc=201956

Iceland: https://www.pettravel.com/immigration/Iceland.cfm;https://www.icelandair.com/support/special-assistance/animal-transportation/

Ireland: https://www.petraveller.com.au/blog/banned-breeds-in-ireland

Lithuania: https://www.pettravel.com/immigration/lithuania.cfm

Norway: https://www.pettravel.com/immigration/Norway.cfm

Poland: https://www.pettravel.com/immigration/poland.cfm

Portugal: https://www.angloinfo.com/how-to/portugal/family/pets-animals/dangerous-dogs ; https://www.dgav.pt/animais/conteudo/animais-de-companhia-2/bem-estar-animal/animais-perigosos-e-potencialmente-perigosos/treino-de-caes-perigosos-e-potencialmente-perigosos/

Romania: https://www.pettravel.com/immigration/Romania.cfm

Spain: https://www.petraveller.com.au/blog/banned-breeds-in-spain

Switzerland: https://www.petraveller.com.au/blog/banned-breeds-in-switzerland

United Kingdom: https://www.pettravel.com/immigration/UnitedKingdom.cfm

Ukraine: https://visitukraine.today/blog/3271/ukraine-officially-allows-taking-pets-to-shelters-under-what-conditions#what-dog-breeds-are-dangerous
