# Supplementary material for: Variations in Canine Behavioural Characteristics across Conventional Breed Clusters and Most Common Breed-Based Public Stereotypes
Source: Animals (Basel). 2024 Sep 17;14(18):2695. doi: 10.3390/ani14182695 (PMC11429495; doi:10.3390/ani14182695)
Supplement: Supplementary file 1 [file animals-14-02695-s001.zip › Table S2 Dog Personality Questionnaire.pdf]

Table S1: Dog Personality Questionnaire (DPQ)

Disagree strongly	Disagree moderately	Disagree slightly	Neither agree nor disagree	Agree slightly	Agree moderately	Agree strongly
1	2	3	4	5	6	7

1. ______ Dog is relaxed when greeting people.
2. ______ Dog behaves aggressively if disturbed or moved when resting.
3. ______ Dog is aloof or indifferent towards other dogs.
4. ______ Dog is destructive.
5. ______ Dog behaves aggressively toward dogs.
6. ______ Dog is anxious
7. ______ Dog loves to be praised.
8. ______ Dog responds aggressively when threatened by another dog (e.g., growled or lunged at, cornered).
9. ______ Dog is bold.
10. ______ Dog is lethargic
11. ______ When off leash, dog comes immediately when called.
12. ______ Dog is shy.
13. ______ Dog behaves aggressively towards unfamiliar people.
14. ______ Dog will work to obtain an object or reward (e.g., ball, treat) that is hidden.
15. ______ Dog likes to chase squirrels, birds, or other small animals.
16. ______ Dog gets bored in play quickly.
17. ______ Dog behaves aggressively when restrained or handled (e.g., groomed).
18. ______ Dog is quick to sneak out through open doors, gates.
19. ______ Dog is confident.
20. ______ Dog is dominant over other dogs.
21. ______ Dog avoids other dogs.
22. ______ Dog catches and kills other animals (e.g., squirrels, rabbits).
23. ______ Dog behaves aggressively when a person (e.g., visitor, delivery person) approaches the house or yard.
24. ______ Dog is easily startled by unexpected contact with objects (e.g., tripping, brushing against a door frame).
25. ______ Dog works at tasks (e.g., getting treats out of a Kong, shredding toys) until entirely finished.
26. ______ Dog is very excitable around cats.
27. ______ Dog is boisterous.
28. ______ Dog behaves fearfully during visits to the veterinarian.
29. ______ When walking on leash, dog tends to pull ahead.
30. ______ Dog behaves fearfully when near crowds of people.
31. ______ Dog enjoys playing with toys.
32. ______ Dog is easily upset when corrected, scolded, or punished.
33. ______ Dog is friendly towards unfamiliar people.
34. ______ Dog is playful with other dogs.
35. ______ Dog seeks companionship from people.
36. ______ Dog behaves submissively (e.g., rolls over, avoids eye contact, licks lips) when greeting other dogs.

Disagree strongly	Disagree moderately	Disagree slightly	Neither agree nor disagree	Agree slightly	Agree moderately	Agree strongly
1	2	3	4	5	6	7

37. ______ Dog is attentive to owner's actions and words.

38. ______ Dog adapts easily to new situations and environments.

39. ______ Dog likes to chase bicycles, joggers, and skateboarders.

40. ______ Dog is curious.

41. ______ Dog guards food or treats from other dogs.

42. ______ Dog is sensitive (and reactive) to pain.

43. ______ Dog behaves aggressively in response to perceived threats from people (e.g., being cornered, having collar reached for).

44. ______ Dog is aloof.

45. ______ Dog is slow to learn new tricks or tasks.

46. ______ Dog is interested in playing tug-o-war with people or dogs.

47. ______ Dog behaves fearfully towards unfamiliar people.

48. ______ Dog is very alert.

49. ______ Dog willingly shares toys with other dogs.

50. ______ Dog is slow to respond to corrections.

51. ______ Dog behaves aggressively during visits to the veterinarian.

52. ______ Dog jumps up on (e.g., in play) and/or mounts other dogs (outside appropriate mating).

53. ______ Dog seeks constant activity.

54. ______ Dog behaves fearfully towards children.

55. ______ Dog is very excitable when visitors arrive.

56. ______ Dog leaves food or objects alone when told to do so.

57. ______ Dog has a tendency to attack (or attempt to attack) other dogs.

58. ______ Dog is quick to recover after being startled or frightened.

59. ______ Dog retrieves objects (e.g., balls, toys, sticks).

60. ______ Dog is friendly towards other dogs.

61. ______ Dog exhibits fearful behaviors when restrained.

62. ______ Dog aggressively guards coveted items (e.g., stolen item, treats, food bowl).

63. ______ Dog is affectionate.

64. ______ Dog ignores commands.

65. ______ Dog behaves aggressively towards cats.

66. ______ Dog behaves fearfully when threatened by other dogs (e.g., growled or lunged at, cornered).

67. ______ Dog follows people around.

68. ______ Dog shows aggression when nervous or fearful.

69. ______ Dog tends to be calm.

70. ______ Dog behaves fearfully towards other dogs.

71. ______ Dog is able to focus on a task in a distracting situation (e.g., loud or busy places, around other dogs).

72. ______ Dog is very excitable around other dogs.

73. ______ Dog behaves aggressively towards children.

74. ______ Dog behaves fearfully when groomed (e.g., nails trimmed, brushed, bathed, ears cleaned).

75. ______ Dog is assertive or pushy with other dogs (e.g., if in a home with other dogs, when greeting).
